# The influence of the polyamine synthesis pathways on Pseudomonas syringae virulence and plant interaction

**DOI:** 10.1099/mic.0.001569

**Published:** 2025-06-10

**Authors:** Leandro Solmi, Franco Rubén Rossi, Fernando Matías Romero, Marcel Bach-Pages, Gail M. Preston, Andrés Gárriz

**Affiliations:** 1 Laboratorio de Fitobacteriología, Instituto Tecnológico de Chascomús (CONICET-UNSAM), Escuela de Bio y Nanotecnologías (UNSAM), Avenida Intendente Marino Km 8.2, Chascomús, CP7130, Buenos Aires, Argentina; 2 Laboratorio de Estrés Biótico y Abiótico en Plantas, Instituto Tecnológico de Chascomús (CONICET-UNSAM), Escuela de Bio y Nanotecnologías (UNSAM), Avenida Intendente Marino Km 8.2, Chascomús, CP7130, Buenos Aires, Argentina; 3 Department of Biology, University of Oxford, South Parks Road, Oxford, OX1 3RB, UK

**Keywords:** polyamines, *Pseudomonas syringae*, putrescine, spermidine

## Abstract

This study investigates the role of polyamine biosynthesis in the pathogenesis of the bacterial phytopathogen *Pseudomonas syringae* pv. *tomato*. Through a comprehensive phenotypic analysis of mutant strains affected in the synthesis of putrescine and spermidine, we reveal a complex interplay between this metabolic pathway and bacterial virulence. Disruption of putrescine synthesis impairs a variety of virulence traits such as motility, biofilm formation, siderophore production, prevention of plant stomatal closure and the functionality of the type III secretion system. This is reversed by reintroducing the deleted genes, but not by the supplementation of culture media with putrescine or apoplastic washing fluids (AWF). Similarly, suppression of spermidine biosynthesis results in a comparable phenotype. However, in this case, the wild-type phenotype is restored by adding spermidine, AWF or expressing the spermidine synthase gene. We conclude that both putrescine and spermidine are important for bacterial virulence and that plant-derived spermidine can partially compensate for bacterial needs. Accordingly, whereas putrescine deficiency leads to a hypovirulent phenotype, spermidine synthesis perturbation does not affect plant colonization. These findings emphasize the critical role of polyamine metabolism in the plant invasion process by bacterial pathogens.

## Introduction

**Fig. 1.**
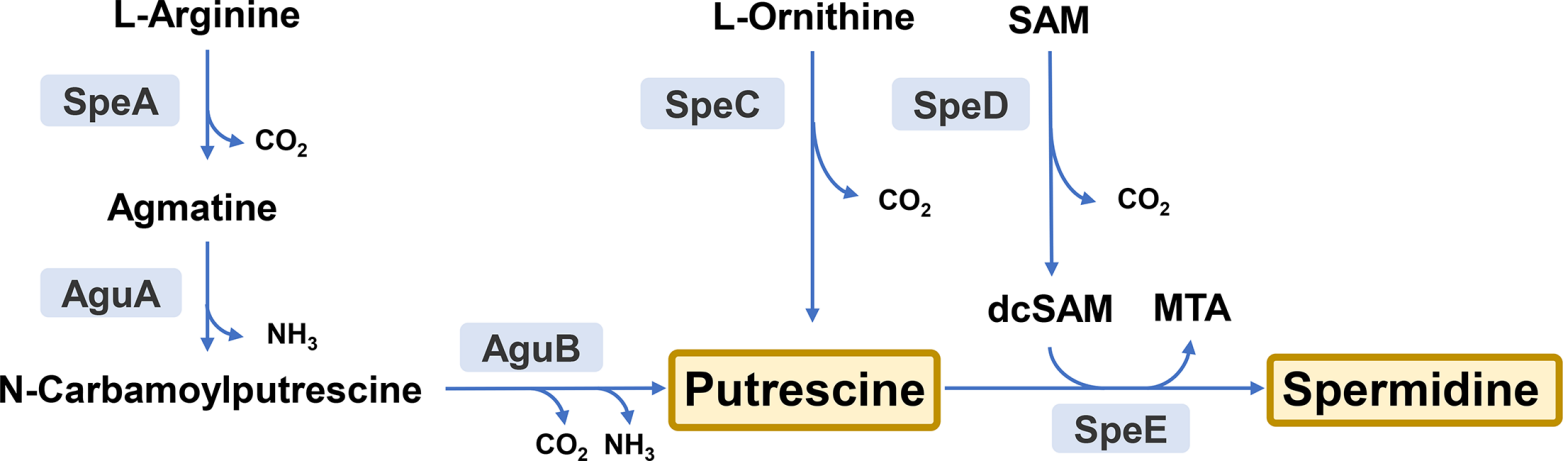
Schematic representation of the putrescine and spermidine synthesis pathways in *Pto*. The main enzymes involved in these metabolic routes are shown in light-blue boxes. SpeA, arginine decarboxylase; SpeC, ornithine decarboxylase; SpeE, spermidine synthase; SpeD, *S*-adenosylmethionine decarboxylase; AguA, agmatine deiminase; AguB, *N*-carbamoyl-putrescine amidohydrolase; SAM, *S*-adenosylmethionine; dcSAM, decarboxylated *S*-adenosylmethionine.

Polyamines are essential for all living organisms, as these small, positively charged polycationic molecules play crucial roles in maintaining membrane integrity and regulating gene transcription and translation. These features stem from their ability to interact with polyanionic molecules such as proteins, nucleic acids and lipids [1,2]. The most abundant polyamines found in bacteria are the diamine putrescine and the triamine spermidine. Bacteria tightly regulate the concentrations of these compounds in both intracellular and extracellular compartments through coordinated adjustments in their synthesis, catabolism and transport pathways. Two distinct routes result in the synthesis of putrescine in bacterial cells (Fig. 1). One of these pathways directly converts the amino acid ornithine into putrescine through the action of the enzyme ornithine decarboxylase (SpeC). Alternatively, putrescine can be generated from the decarboxylation of arginine by the enzyme arginine decarboxylase (SpeA) and two subsequent enzymatic steps with agmatine and *N*-carbamoyl-putrescine serving as intermediate molecules. In turn, spermidine is then formed from putrescine through the action of the enzyme spermidine synthase (SpeE), which uses decarboxylated *S*-adenosylmethionine as an aminopropyl donor [3].

The generation and study of mutant strains lacking the genes mentioned above have convincingly shown that polyamine synthesis influences bacterial growth and virulence [4,10]. Even though these studies primarily focused on human pathogens such as *Escherichia coli*, *Pseudomonas aeruginosa* and *Salmonella enterica*, recent evidence underscores the importance of polyamines in plant pathogens as well. For instance, Lowe-Power *et al.* [11] demonstrated that a mutant strain of *Ralstonia solanacearum* incapable of synthesizing polyamines exhibits reduced virulence in tomato plants. Apparently, the reduction in the production and secretion of putrescine affects the survival of bacterial cells under the oxidative conditions imposed by the elicitation of the plant immunity mechanisms. Deletion of the *speA* gene in *Dickeya zeae* also reduces virulence in rice seeds [12]. Interestingly, the lack of *speA* in this species has a greater impact on a strain where the genes coding for polyamine transporters were deleted, suggesting that the synthesis and transport of polyamines act coordinately to fulfil bacterial requirements. Additional research should be conducted to elucidate the mechanisms underlying the connection between polyamine functions and pathogenesis in bacterial plant pathogens.

The bacterial species *Pseudomonas syringae* is regarded as one of the most formidable plant pathogens, owing to its wide distribution and its ability to infect a vast array of plant hosts [13]. With over 60 identified pathovars within this species, it shows an impressive adaptability to diverse environmental niches. Among these, the strain DC3000 belonging to pv. *tomato* (referred to as *Pto*) has garnered the most attention to date, as its ability to infect both tomato and *Arabidopsis thaliana* renders it an invaluable model for exploring the intricate dynamics of plant–pathogen interactions. Indeed, a substantial portion of our current understanding regarding the molecular interplay between plants and bacterial pathogens has been obtained from investigations centred around this pathosystem.

In a previous study, we generated and characterized *Pto* deletion strains lacking genes involved in the polyamine biosynthetic pathway to better understand their role in growth and oxidative stress tolerance [14]. This work demonstrated that *speA* and *speC* function redundantly in putrescine production, as single deletions had no noticeable phenotypic effects. Only the simultaneous deletion of both genes significantly impaired growth and stress tolerance, a defect that was reversed by reintroducing either gene but not by polyamine supplementation, suggesting a potential defect in polyamine uptake. In contrast, disruption of spermidine synthesis in a *ΔspeE* mutant had minor effects on growth but enhanced stress tolerance, which was reduced upon *speE* reintroduction or spermidine supplementation. These findings revealed a previously unknown link between polyamine homeostasis and stress tolerance.

Our previous work also suggests that the synthesis of polyamines might play a significant role in bacterial pathogenesis. In this regard, we showed the induction of this pathway during plant colonization and the accumulation of putrescine in the plant apoplast [15]. Furthermore, we conducted a meta-analysis of publicly accessible transcriptomic data on phytopathogenic bacteria that demonstrated that genes from this metabolic route are induced during the early stages of infection and that they are suppressed by plant immunity mechanisms [16].

In the present study, we further investigated the roles of polyamines in *Pto* and their significance in plant invasion and pathogenesis. The experiments described in this work revealed that the deletion of genes encoding putrescine- and spermidine-synthesizing enzymes significantly impacts various traits essential for plant invasion, including growth, motility, siderophore production and evasion of stomatal closure. However, these alterations do not consistently affect virulence within the plant environment. Therefore, we conclude that while polyamine synthesis is vital for initiating pathogenesis and facilitating plant invasion, polyamines produced by the host might fulfil the pathogen’s requirements to some extent. The implications of these findings are further discussed.

## Methods

### Biological material and growth conditions

Bacterial strains (Table 1) were routinely cultured on LB agar plates at 28 °C. Bacterial inocula were prepared from cells grown in liquid LB at the same temperature with shaking (160 r.p.m.) for 18 h. These cells were washed two times with 10 mM MgCl_2_ by centrifugation-resuspension cycles and diluted to the working OD_600_. Minimal media M9 and Hrp-inducing medium (HIM) were prepared as described by Miller [17] and Kim *et al.* [18], respectively.

**Table 1.** Bacterial strains used in this study

Strain	Relevant characteristics	Reference
*Pto*	*P. syringae* pv. *tomato* DC3000, Rif^R^	[53]
*corS*	*Pto corS::aphA;* Rif^R^ Km^R^	[34]
*ΔspeE*	*Pto* unmarked deletion mutant lacking *speE* gene, Rif^R^	[14]
*ΔspeA/speC*	*Pto* unmarked deletion mutant lacking *speA* and *speC* genes, Rif^R^	[14]
*ΔspeE (speE*)	*ΔspeE* complemented with a pBBR1MCS-5 derivative carrying the *speE* gene with its native promoter, Gm^R^-Rif^R^	[14]
*ΔspeA/speC (speA*)	*ΔspeA/speC* complemented with a pBBR1MCS-5 derivative carrying the *speA* gene with its native promoter, Gm^R^-Rif^R^	[14]
*ΔspeA/speC (speC*)	*ΔspeA/speC* complemented with a pBBR1MCS-5 derivative carrying the *speC* gene with its native promoter, Gm^R^-Rif^R^	[14]
*Pto-GFPuv*	*Pto* carrying pDSK-GFPuv	[14]
*ΔspeE-GFPuv*	*ΔspeE* carrying pDSK-GFPuv	[14]
*ΔspeA/speC-GFPuv*	*ΔspeA/speC* carrying pDSK-GFPuv	[14]

Seeds of *A. thaliana* ecotype Columbia-0 were germinated in a perlite–soil–sand mix (1:1:1) and cultivated in a growth chamber with a 10 h day/14 h night photoperiod (photon flux density of 100 µmol m s^−1^ provided by cool-white fluorescent lamps), with 22±2 °C and 55±5% temperature and relative humidity, respectively. In turn, seeds of *Nicotiana benthamiana* were germinated in the described mix, changing the light provision to a 16 h day/8 h night photoperiod (photon flux density of 200 µmol m s^−1^), with 24±2 °C and 65±5% temperature and relative humidity, respectively. The plants were regularly watered with half-strength Hoagland’s nutrient solution [19]. Six-week-old *A. thaliana* plants or fully expanded leaves detached from 6-week-old *N. benthamiana* plants were used. Alternatively, *A. thaliana* seeds were disinfected with 70% (v/v) ethanol for 2 min, followed by 15 min in 5% (w/v) sodium hypochlorite and 0.1% (w/v) SDS solution and a subsequent washing step in sterile distilled water. Seeds were then dispensed in Petri dishes containing MS agar medium (Murashige and Skoog, Sigma-Aldrich) and incubated in a plant growth chamber for 2 weeks.

### Quantification of biofilm production

Biofilm quantification was carried out using the methodology described by Qu *et al.* [20] with some modifications. Briefly, cells were grown overnight at 28 °C with constant shaking in LB medium supplemented with rifampin (50 µg ml^−1^) and gentamicin (10 µg ml^−1^) when needed. Subsequently, the cells were washed twice with 1X PBS buffer, and 200 µl of LB or M9 medium was inoculated into wells of X96 multi-well plates at a final OD_600_ of 0.1 (~1×10^8^ cells ml^−1^). The plates were incubated statically at 28 °C for 24 h. After the incubation period, bacterial growth was quantified by measuring absorbance at 600 nm using a Synergy H1 Hybrid Multi-Mode Microplate Reader (BioTek Instruments, Inc.). Cells were then removed by inverting the plate onto a waste collector, and the wells were washed twice with 200 µl of 1X PBS buffer. Briefly, 200 µl of 0.1% crystal violet solution was added and left for 20 min at room temperature to allow staining of the bacterial biofilm. Finally, each well was washed three times with 200 µl of 1X PBS buffer, and stained biofilm was solubilized by adding 200 µl of 96% ethanol (v/v). The absorbance of the dissolved crystal violet (590 nm) in the ethanol solution was measured and the values were normalized to bacterial growth.

### Cell motility assay

The methodology originally described by Adler [21] was adapted to evaluate cell motility. Thus, *Pto* strains were cultured overnight at 28 °C in LB medium with constant shaking. Subsequently, cells were washed with 1X PBS buffer and its concentration was quantified by measuring the OD at 600 nm. Glass test tubes containing 2 ml of either LB or M9 medium were inoculated to reach a final OD_600_=0.1. In parallel, 40 µl of uninoculated LB or M9 medium was placed inside glass capillaries (1.40 mm internal diameter) and sealed at the top. The capillaries were placed inside the test tubes containing cell cultures in the same medium and incubated statically at 22 °C for 4 h. Then, the capillaries were removed and the number of c.f.u. was quantified from a 20 µl sample of their contents.

Alternatively, the same procedure was performed to grow the cells and obtain the inoculum but using WT, *ΔspeE* and *ΔspeA/speC* versions transformed with the pDSK-GFPuv plasmid. Tubes with 2 ml of LB or M9 were inoculated at a cell concentration of 0.1 at OD_600_, incubated at 22 °C for 6 h, and then samples from these cultures were observed under a fluorescence microscope. The coverslip was sealed with nail polish to prevent dehydration. At least five samples from each tube were observed and recorded, and at least five cells per sample were tracked. The distance travelled by the cells was measured over 60 s to calculate their speed.

### Quantification of pyoverdine secretion

The estimation of extracellular pyoverdine was carried out as described by Barrientos-Moreno *et al.* [22] with minor modifications. *Pto* and its mutant strains were cultured in M9 medium for 18 h, and cell growth was measured by OD at 600 nm. When indicated, cultures were supplemented with 50 µM FeCl_3_ and 2 mM putrescine or spermidine. Then, 800 µl of each culture was centrifuged at 8,000 ***g*** for 5 min to obtain cell-free supernatants. The fluorescence derived from pyoverdine in this fraction was quantified using a Synergy H1 Hybrid Multi-Mode Microplate Reader (BioTek Instruments, Inc.), recording the emission at 455 nm with an excitation wavelength of 398 nm. The fluorescence readings were corrected based on the absorbance at 600 nm.

### Stomatal opening assay

The ability of bacteria to inhibit stomatal closure in *A. thaliana* was studied using a method adapted from Chitrakar and Melotto [23]. Adult *A. thaliana* plants were kept well-hydrated in trays, covered with film and exposed to light for at least 4 h to induce stomatal opening. Fully expanded leaves from each plant were cut and submerged in 10 mM MgCl_2_, either uninoculated (control) or with a final concentration of 1×10^8^ cells ml^−1^ of bacteria (~OD_600_=0.1). After 4 h of incubation at 25 °C in the presence of light, the leaves were removed from the solution, completely dried with absorbent paper, and the abaxial epidermal layer was removed with surgical tweezers. Epidermal cells were stained with a 20 µM propidium iodide solution for 10 min, then washed three times with 10 mM MgCl_2_ and mounted on slides. Finally, samples were visualized under a fluorescence microscope (ZEISS Axio Observer, Carl Zeiss Inc.) using a 40× objective with an excitation wavelength of 453 nm and an emission filter of 579–694 nm. The stomatal opening index (µm width/µm length) was calculated by observing at least 30 stomata per leaf.

### Plant inoculation

Fully developed *N. benthamiana* leaves were inoculated with ~200 µl of three different cell suspensions (1×10^6^, 1×10^7^ and 1×10^8^ cells ml^−1^) using a sterile 1 ml syringe without a needle, and the infiltration area was delimited with a permanent marker. Plants were incubated for 24 h under the described growth conditions, after which the degree of lesion in the infiltrated areas was assessed.

*A. thaliana* seedlings were inoculated using the flooding method. For this purpose, culture plates were flooded with 40 ml of 10 mM MgCl_2_+0.025% Silwet L-77, with or without bacterial cells (5×10^6^ cells ml^−1^) and incubated for 3 min at room temperature, as reported by Ishiga and Ichinose [24]. The solutions were carefully removed, and the plates were left to dry in a laminar flow hood for 10 min and then incubated at normal growth conditions. At 24 and 48 h post-inoculation, the shoots were collected (three per biological sample) and placed in 1.5 ml Eppendorf tubes. Each biological sample was weighed, washed with 10 mM MgCl_2_ and surface-sterilized with 5% H_2_O_2_ for 3 min. After three subsequent washes with sterile 10 mM MgCl_2_, the plant tissue was homogenized in 500 µl of the same solution using a disruptor and 2 mm diameter steel beads. The samples were then centrifuged at 3,000 ***g*** for 3 min, and serial dilutions of the supernatant were performed to quantify c.f.u. The values obtained were normalized to the initial fresh weight of each sample.

Alternatively, *A. thaliana* adult plants were incubated in plastic trays for at least 2 h covered with film. Two leaves (second–third pair of true leaves) from each plant were selected and solutions of sterile 10 mM MgCl_2_ (control) or a bacterial suspension (final concentration of 2×10^6^ cells ml^−1^) were used to completely infiltrate the leaves using 1 ml syringes. The plants were later incubated under the same growth conditions for 24, 48, 72 and 96 h. Samples were taken at each time point using a 0.5 cm diameter punch. Each biological sample consisted of four discs taken from two leaves. The discs were placed in 1.5 ml Eppendorf tubes and homogenized in 250 µl of sterile 10 mM MgCl_2_ using a tissue disruptor. Serial dilutions of the homogenates were made after low-speed centrifugation, plated on LB medium supplemented with rifampin and incubated for 48 h to count c.f.u.

### Quantification of H_2_O_2_ accumulation in leaves

Following the same procedure described in the previous section, *N. benthamiana* leaves were inoculated with bacterial suspensions at a final concentration of 1×10^7^ cells ml^−1^ or 10 mM MgCl_2_ as a control. The inoculated area was marked, and the plants were incubated for 18 h under the mentioned growth conditions. To assess H_2_O_2_ accumulation, the protocol described by Bach-Pages and Preston (2018) was followed. Briefly, the leaves were cut, placed in 50 ml tubes (one leaf per tube) and submerged in a 0.1% (w/v) DAB-HCl solution (3,3-diaminobenzidine-HCl, pH 3.8). After incubating the tubes at room temperature for 8 h, the staining solution was removed and the leaves were washed three times with 10 mM MgCl_2_. Then, 40 ml of 96% (v/v) ethanol was added to each tube and heated at 65 °C in a water bath for 2 h. The solution was replaced with 75% (v/v) ethanol and tubes incubated at room temperature. Once the leaves were completely decolourized, a white light transilluminator was used to visualize and take pictures.

### Extraction of apoplastic washing fluids from *A. thaliana* and quantification of polyamines

The extraction of apoplastic fluids was carried out according to the method described by O'Leary *et al.* [25]. Briefly, adult *A. thaliana* plants were cut at the base of the rosette, washed twice with distilled water, dried and submerged in distilled water. They were later subjected to vacuum using a pump (at a pressure of 500 mm Hg) until the leaves were completely infiltrated. Subsequently, plants were removed, dried and inserted into 20 ml syringes placed into 50 ml tubes and centrifuged at 1,500 ***g*** for 15 min at 4 °C. The resulting liquid was stored at 4 °C until use. Chlorophyll content in the apoplastic fluid was measured spectrophotometrically in relation to its content in total plant material in order to assess contamination with cytoplasmic components. Based on the values of chlorophyll concentrations, we determined that under our experimental conditions, the cytosolic contamination of the apoplastic washing fluids (AWF) was under 1%. Free polyamines in these samples were quantified as previously described in [15].

### Gene expression profiling

Bacterial strains were grown overnight in LB medium, later centrifuged at 8,000 ***g*** for 5 min and washed twice with 10 mM MgCl_2_. Subsequently, 4 ml of LB or HIM media were inoculated at a final concentration of 5×10^7^ cells ml^−1^ and incubated for 6 h at 28 °C with constant shaking (160 r.p.m.). Cultures were centrifuged at 12,000 ***g*** for 10 min at 4 °C. The pellets were washed once with 10 mM MgCl_2_ and frozen at −80 °C until processing. RNA extraction was performed using TRANSZOL reagent (TRANS®) following the manufacturer’s instructions, with an additional chloroform extraction step and two 75% ethanol washing steps. RNA integrity was assessed by electrophoresis on 1.2% (w/v) agarose gels, and nucleic acid concentration was quantified by absorbance at 230 nm using a Take3 plate (BioTek®). RNA samples were treated with TURBO DNase I (Invitrogen™) to degrade any potential genomic DNA contamination. Subsequently, cDNA was generated by adding 1.2 µg of RNA to the following mixture: 1 µl of random hexamers (dN_6_), 1 µl of 10 mM dNTPs, 1 µl of MMLV reverse transcriptase (200 U µl^−1^), 5 µl of 5X reaction buffer (250 mM Tris/HCl, 375 mM KCl, 15 mM MgCl_2_ and 50 mM DTT, pH 8.3) and distilled water up to a final volume of 25 µl. The reaction mixture was incubated for 1 h at 37 °C. Quantitative real-time PCR (qRT-PCR) was performed using the primers and protocol as described in Vilas *et al*. [15].

### Statistics

Each experiment was conducted at least twice with similar results, and representative experiments are shown. Each treatment included at least five biological replicates. The results are presented as the means±sd. Data were analysed by Student’s t-test or ANOVA followed by post-hoc comparisons by Tukey’s test. The qRT-PCR results were analyzed following the method described by Pfaffl *et al.* [26].

## Results

### Polyamines regulate both the motile and sessile states and facilitate tissue invasion

The transition to a multi-cellular sessile state and the formation of biofilms is crucial for bacterial survival, enabling them to withstand harsh environmental conditions on plant surfaces and evade plant defence responses [27,28]. Therefore, controlling this phenotypic switch is important for bacterial survival and proliferation of bacteria in plant tissues. Given that polyamines have been implicated in regulating motile and static states across various bacterial species [29,32], we first looked into whether this is also applicable to *Pto* by examining the phenotype of a single mutant in the spermidine synthase gene (*ΔspeE*) and that of a double mutant where both putrescine synthetic pathways are blocked (*ΔspeA/speC*).

**Fig. 2.**
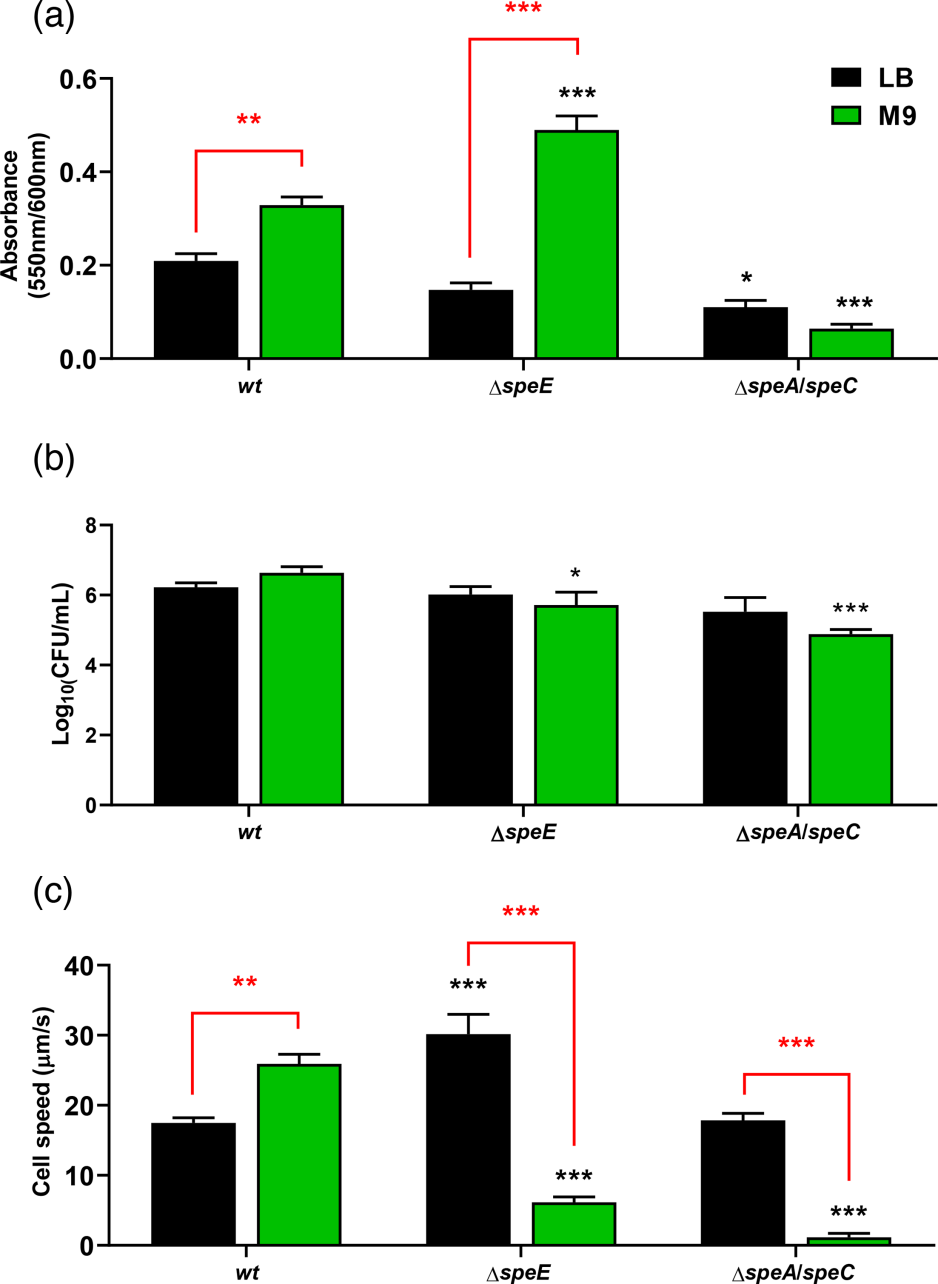
Effect of polyamine synthesis deficiency on biofilm production and cell motility in *Pto*. (**a**) The formation of biofilms in the indicated strains was carried out using the crystal violet staining method. Thus, cells were grown statically in LB or M9 medium for 24 h at 28 °C in multi-well plates. Subsequently, each well was washed and staining was performed as described in Methods. The absorbance at 550 nm was measured to estimate biofilm production, and values were normalized to cell growth as determined by the absorbance at 600 nm. (**b**) Cell swimming ability was assessed in liquid cultures at 22 °C. The values represent the c.f.u. counts (corrected by base-10 logarithm) found inside capillaries submerged in the indicated cultures for 4 h. (**c**) Cell speed was assessed by tracking cell trajectories over 60 s using a fluorescence microscope. Statistical analysis of the data was performed using ANOVA followed by Tukey’s post-hoc test for multiple comparisons. Significant differences between strains cultured on the same growth medium, or between different growth media for the same strain (indicated in red brackets), are presented as *P<0.05, **P<0.01, ***P<0.001. The assays were performed in triplicate with similar results.

As depicted in Fig. 2(a), *Pto* shows a tendency to assemble larger biofilms in the minimal medium M9 in comparison to LB, which is a complex medium containing amino acids and other metabolites derived from yeast extract, including ornithine, putrescine, spermidine, spermine and *S*-adenosylmethionine. The Δ*speE* strain exhibits a greater capacity to build these structures than the *wt* when growing in M9, but no differences were observed between the *wt* and the *ΔspeE* strain in LB. Biofilm formation, however, was significantly diminished in the double mutant Δ*speA*/*speC* regardless of the culture medium used. Importantly, because this strain exhibits severe growth rate impairment, we standardized the results based on their ODs at 600 nm prior to the staining procedure to ensure accurate comparisons. Complementation of the *ΔspeE* strain with *speE* reinstated the *wt* phenotype (Fig. S1, available in the online Supplementary Material). Although complementation with *speA* or *speC* enhanced biofilm structuration by *ΔspeA/speC*, no differences were seen in biofilm formation between the two media, in contrast with the *wt*.

These findings are intriguing since we have previously demonstrated that the effects on growth rate and polyamine levels in *ΔspeA*/*speC* were recovered upon its complementation with either of these genes [14]. Therefore, it is probable that putrescine incorporation plays an active and quantitative role in biofilm formation, which cannot be fully restored when one of the pathways leading to its production is blocked. This is also supported by the increased biofilm formation shown by the *ΔspeE* strain, which, by blocking the conversion of putrescine to spermidine, could also contribute to the accumulation of putrescine provided that the medium contains this metabolite. This is a hypothesis that should be confirmed by examining the bacteria’s capacity to incorporate polyamines.

In turn, the swimming phenotype of the *wt* strain was not affected by the availability of nutrients (Fig. 2b). When cultured in rich medium, the motility rates of *ΔspeE* and *ΔspeA/speC* were comparable to the *wt*; however, in M9 liquid cultures, both mutant strains exhibited a slight yet consistent decrease in their swimming capacity. In this case, we measured swimming over a brief incubation period (4 h) so that the impact of a growth defect may be minimized, although it cannot be entirely ruled out. Therefore, we also used fluorescence microscopy to measure cell velocity in order to better understand cell motility in mutant strains (Fig. 2c). Our findings showed that the *wt* strain’s swimming speed is influenced by nutrition availability, as it significantly increases under nutrient restriction. In contrast to the wild-type, the *ΔspeE* strain surprisingly demonstrated a considerably faster swimming speed in LB. Finally, swimming speeds of both mutant strains were much slower than the wild-type in M9, which is in line with the findings in Fig. 2(b). The reduced motility of the *ΔspeE* mutant may help explain its enhanced ability to form biofilms. However, the lower motility of the *ΔspeA/speC* double mutant does not result in increased biofilm formation, likely due to the additional impact of its impaired growth rate.

**Fig. 3.**
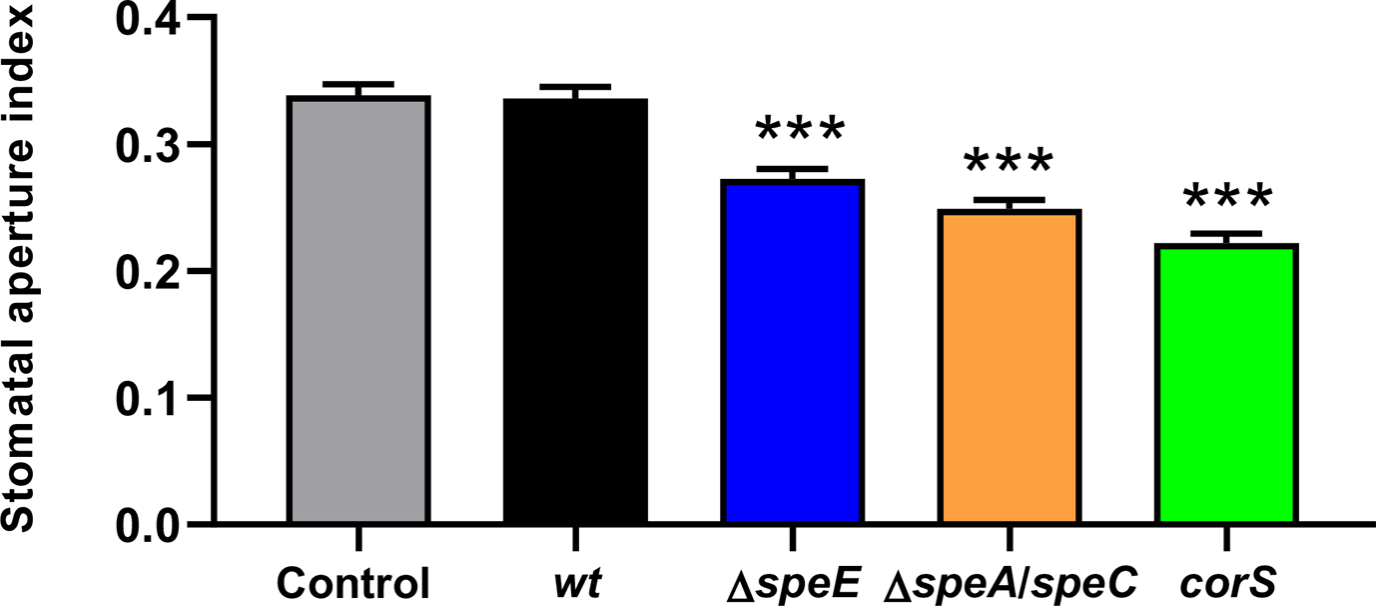
The synthesis of polyamines is important for the inhibition of stomatal closure. Plant leaves were exposed for 4 h to a cell suspension of *wt*, *∆speE*, *∆speA/speC* and *corS*. Then, the abaxial epidermal layers of the leaves were removed and stained with 10 µM propidium iodide, and the stomatal aperture index was quantified using fluorescence microscopy. The data were analysed using ANOVA, followed by Tukey’s post-hoc test for multiple comparisons. Significant differences between treatments and controls are presented as ***P<0.001. The assay was performed in triplicate with similar results.

Prevention of stomatal closure to facilitate bacterial entry into plant cells is one of the most important pathogenic determinants in *Pto*, which is mainly mediated by the production of the polyketide toxin coronatine [33]. To assess the ability of the *wt* and mutant strains to maintain the stomata in an open state, we submerged *A. thaliana* leaves in suspensions of the respective bacterial strains and quantified stomatal opening using fluorescence microscopy. As depicted in Fig. 3, stomata in leaves treated with the *wt* strain showed aperture index values comparable to those determined in control leaves incubated in MgCl_2_, but both the *ΔspeE* and *ΔspeA/speC* strains failed to prevent stomatal closure. A mutant unable to produce coronatine (*corS* [34]) was used for comparison purposes.

### Putrescine and spermidine synthesis are required for optimal pyoverdine production

In bacteria, the production of a series of small iron-binding molecules known as siderophores facilitates scavenging of this element from the environment. *Pseudomonas* species such as *Pto* produce pyoverdines under low-iron conditions, a family of fluorescent siderophores that are considered a virulence factor [35]. It has been demonstrated that pyoverdine production is abolished in *Pseudomonas putida* when the synthesis of arginine is perturbed and that it is rescued by the addition of this amino acid as well as spermidine [22]. Therefore, we speculated that a link between polyamine synthesis and siderophore production may exist in *Pto*. With this in mind, we evaluated and compared the fluorescence derived from pyoverdine production between the *wt* and mutant strains.

**Fig. 4.**
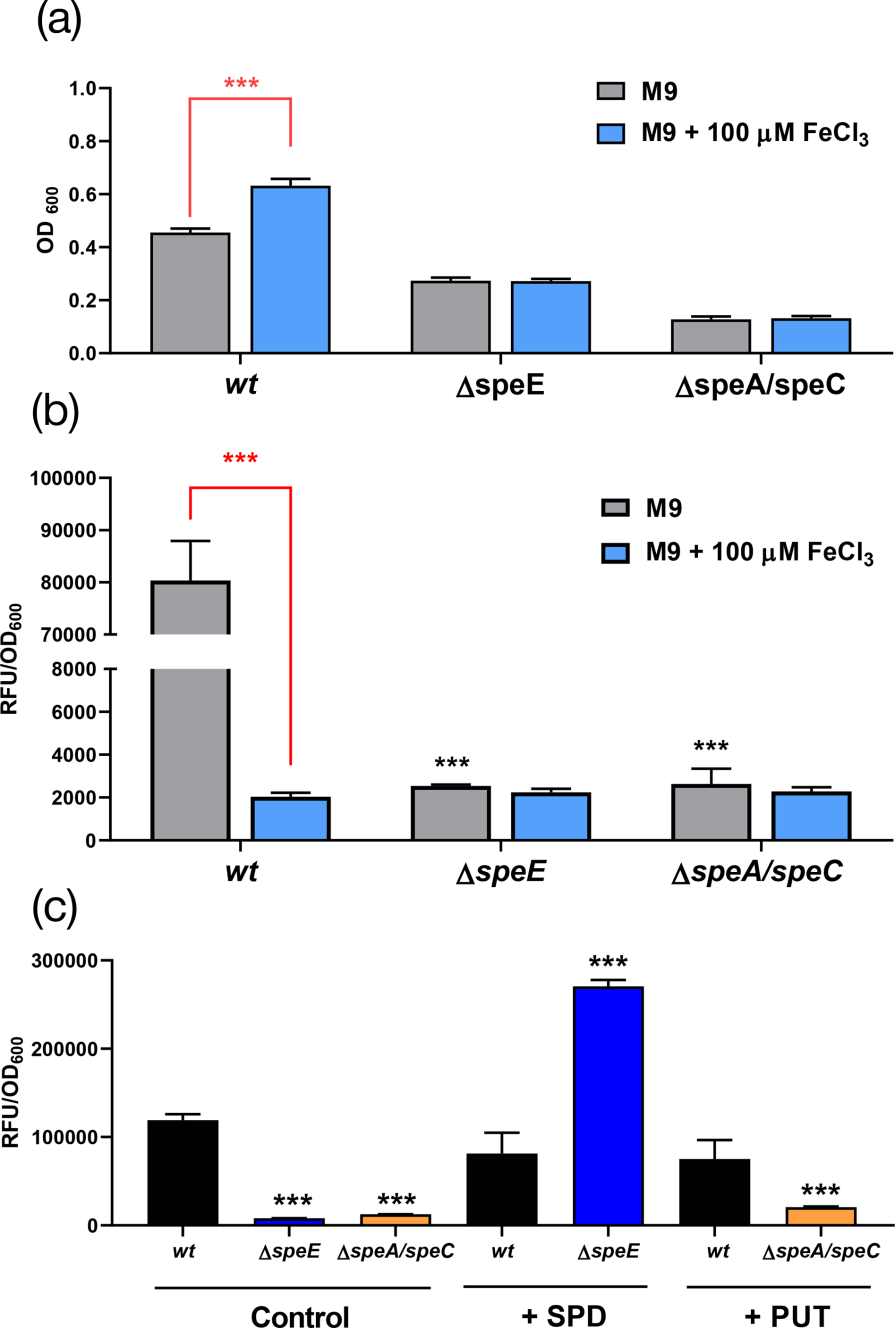
Perturbation in the synthesis of polyamines affects pyoverdine production. (**a**) Cells were cultivated at 28 °C in M9, either under control conditions or supplemented with 100 µM FeCl_3_. OD_600_ was determined after 4 h to estimate bacterial growth. (**b**) Assessment of relative fluorescence units (RFU, excitation 398 nm/emission 455 nm) in cell-free supernatants of *Pto* strains cultured under the same conditions described in (**a)**. The supernatants were collected by centrifugation, and the fluorescence levels were normalized to the estimated cell concentration as measured by OD_600_. The same procedure was followed in (**c)**, but cultures were supplemented with polyamines at a final concentration of 2 mM. Data were analysed by ANOVA, followed by Tukey’s post-hoc test for multiple comparisons. Significant differences between strains are presented as ***P<0.001, whereas the differences between different treatments for the same strain are represented in red brackets.

The addition of FeCl_3_ increased the growth rate of the *wt* strain and significantly decreased the fluorescence of the culture media, as shown in Fig. 4(a, b) (first two bars). This aligns with the fact that iron is a limiting nutrient in M9 and its presence can inhibit the synthesis of pyoverdine. Unlike the *wt* strain, neither the Δ*speE* nor the Δ*speA/speC* strains showed increased growth in FeCl_3_-amended media, which could be related to an impairment in their ability to acquire iron (Fig. 4a). Consistently, the fluorescence of the culture media remained notably low for the mutant strains under all tested growth conditions (Fig. 4b), suggesting that pyoverdine production is hindered when putrescine and/or spermidine synthesis is limited. Supplementation with spermidine or putrescine did not induce pyoverdine production in the *wt* strain under low-iron conditions. However, spermidine could restore pyoverdine generation in *ΔspeE*, while putrescine did not promote its formation in *ΔspeA/speC* (Fig. 4c). Importantly, pyoverdine-derived fluorescence was restored in *ΔspeA/speC* when complemented with either *speA* or *speC*, as well as in *ΔspeE* by the expression of *speE* (Fig. S2). This indicates that while putrescine and spermidine synthesis is essential for optimal pyoverdine production, only the presence of the latter in the extracellular space can compensate for disruptions in the synthetic pathway. Consequently, it is possible that *ΔspeE* (but not *ΔspeA/speC*) could still produce pyoverdine in the plant environment where spermidine is abundant [15,36].

### Polyamine perturbation affects the functioning of the type III secretion system

The proper assembly of the type III secretion system (T3SS) is crucial for the secretion and translocation of bacterial effectors into the plant cells, which help modulate plant defence mechanisms. A simple way to assess the overall functioning of this protein system in *Pto* is through its inoculation in the model plant *N. benthamiana*, which has the ability to identify T3SS-secreted bacterial effectors (such as HopQ1-1) and trigger cell death associated with a hypersensitive response (HR) [37]. Thus, we infiltrated serial dilutions of *wt* and mutant strains in leaves of this species and visually monitored the development of symptoms.

**Fig. 5.**
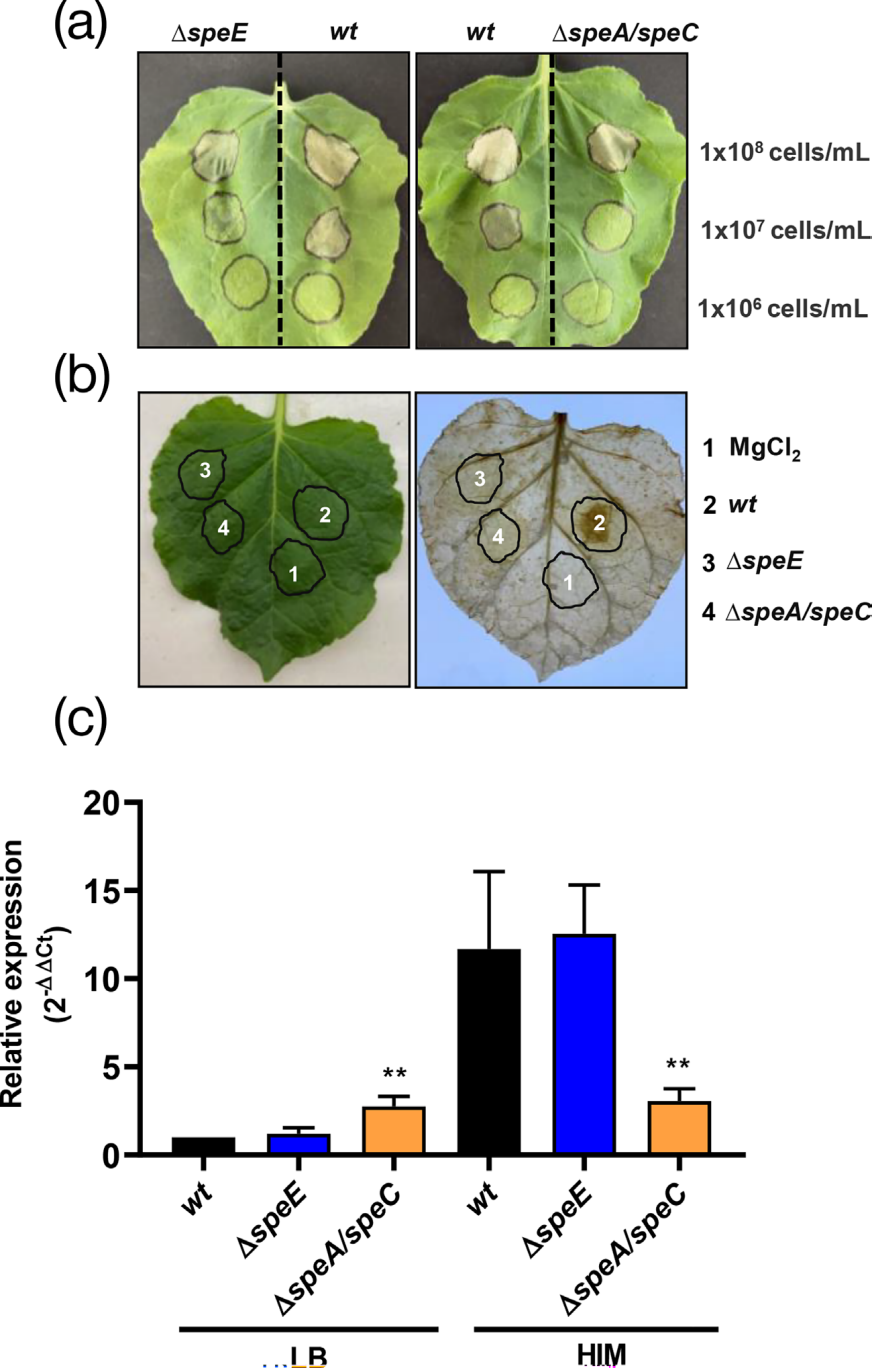
Effect of polyamine synthesis perturbation on the functionality of the T3SS in *Pto*. (**a**) Fully expanded leaves of *N. benthamiana* were inoculated using sterile syringes with different cell suspensions of *wt*, *∆speA/speC* or *∆speE*. 10 mM MgCl_2_ was used as a negative control. After treatment, the leaves were maintained under the same conditions for 24 h and photographed. (**b**) The same process described in (**a) **was followed using suspensions of 1×10^7^ cells ml^−1^, and after 18 h of incubation, the leaves were stained with DAB to evidence H_2_O_2_ accumulation as detailed in Methods. (**c**) Expression of the bacterial gene *hopA* in *wt* and polyamine synthesis mutant strains. The cells were cultured in LB and HIM media for 6 h and harvested by centrifugation. Transcript levels were normalized to the average signal intensity in LB, which was assigned a value of 1. Results are the mean of five replicates+standard error, and statistically significant differences in gene expression between mutant strains and *wt* are shown as **P<0.01. The assay was performed in triplicate with similar results.

As illustrated in Fig. 5(a), both mutant strains led to reduced degrees of cell death at the inoculation site compared to the *wt*. The production of hydrogen peroxide associated with the induction of HR was reduced in these cases, as evaluated by the DAB staining method (Fig. 5b). In addition to this, the expression of the gene encoding the effector protein HopA was significantly induced in cells of the *wt* and *ΔspeE* when cultured in the HIM, but *hopA* expression was significantly lower in the *ΔspeA/speC* mutant strain (Fig. 5c). On this basis, we concluded that polyamine synthesis plays an important role in the functionality of the T3SS.

Altogether, the findings described above align with a model where putrescine and spermidine synthesis are crucial for bacterial swimming, stomatal closure prevention, siderophore production and effector translocation. In subsequent experiments, we examined the impact of polyamine synthesis disruption on the bacterial capacity to colonize plant tissues.

### An intact polyamine biosynthesis pathway in *Pto* is vital in plant invasion

In an earlier study, we analysed publicly accessible transcriptome data published for *P. syringae* in order to gain a preliminary understanding of how the metabolism of polyamines is regulated during plant invasion [16]. This study demonstrated that polyamine production is activated during the first stage of bacterial colonization of host tissues, suggesting a critical function for this route in bacterial pathogenicity. To support this hypothesis, we evaluated in the present work the infectivity in *A. thaliana* of the mutant strains impaired in polyamine synthesis.

**Fig. 6.**
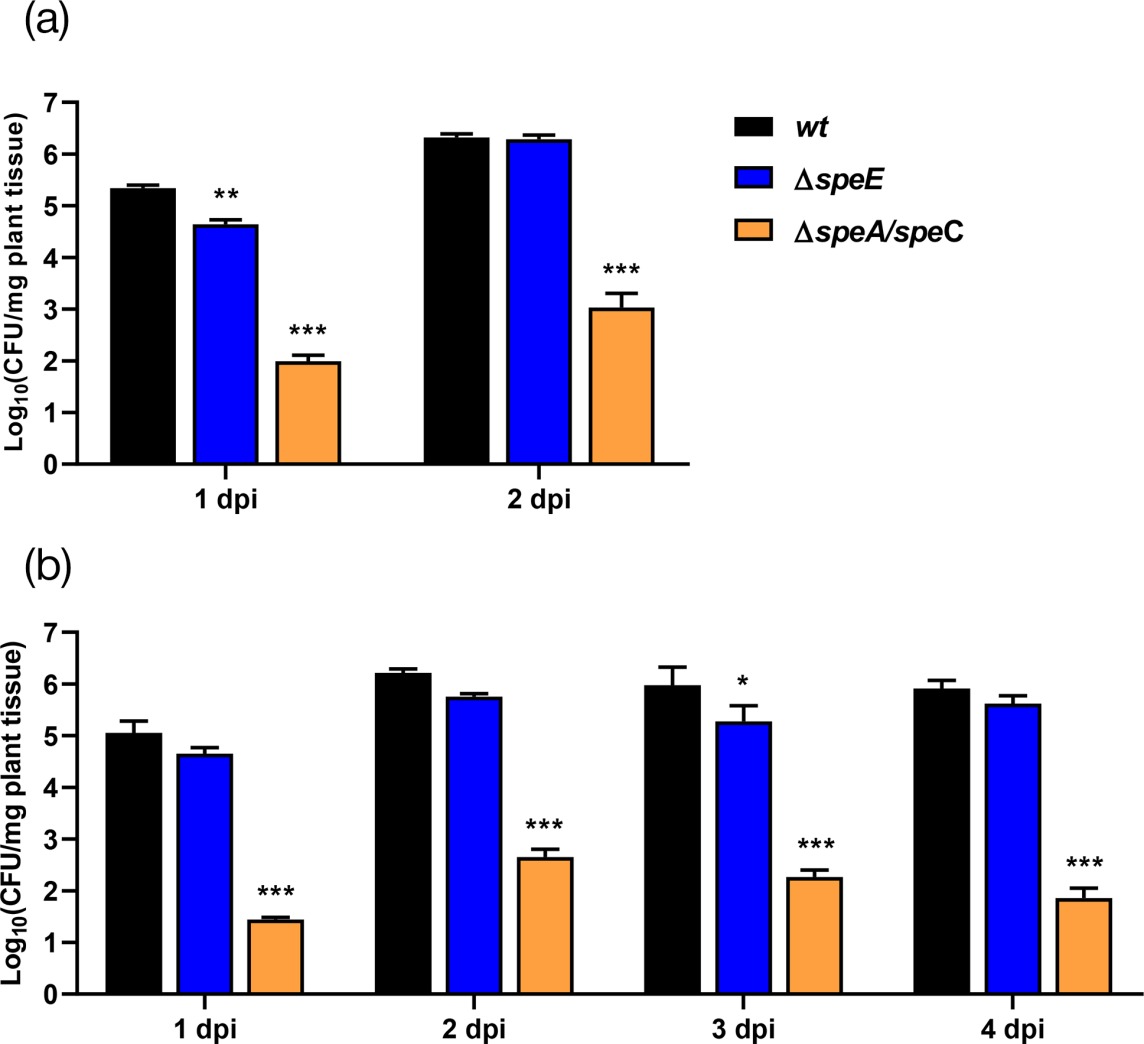
Plant colonization is impaired in *Pto* mutant strains. *A. thaliana* seedlings or plants were inoculated using the (a) flooding method and (b) by infiltration, respectively. C.f.u. in plant tissues on different days after inoculation are presented. The data were analysed using ANOVA, followed by Tukey’s post-hoc test for multiple comparisons. Significant differences between the mutant strains and the *wt* at the same time point are presented as *P<0.05, **P<0.01, ***P<0.001. The assay was performed in triplicate with similar results.

We first assessed virulence in *A. thaliana* seedlings through flooding, since this method takes into account the bacteria’s capacity to enter plant tissues and proliferate inside them. As expected, the *wt* population increased at 1 and 2 days post-inoculation (Fig. 6a). After 1 day of treatment, the c.f.u. counted from seedlings inoculated with *ΔspeE* were marginally but significantly lower; however, the levels recovered and were similar to the *wt* thereafter. In contrast, the *ΔspeA/speC* strain showed a drastic decrease in virulence over the course of the experiment.

We subsequently inoculated fully developed leaves from adult plants by the syringe-infiltration method. In this case, plant entrance is bypassed, and the procedure primarily evaluates the bacteria’s capacity to proliferate and thrive within the host. We observed similar c.f.u. counts in plants infected by either the *wt* or *ΔspeE* throughout the experiment, whereas significantly lower c.f.u. counts were evident when the *ΔspeA/speC* strain was assessed (Fig. 6b).

**Fig. 7.**
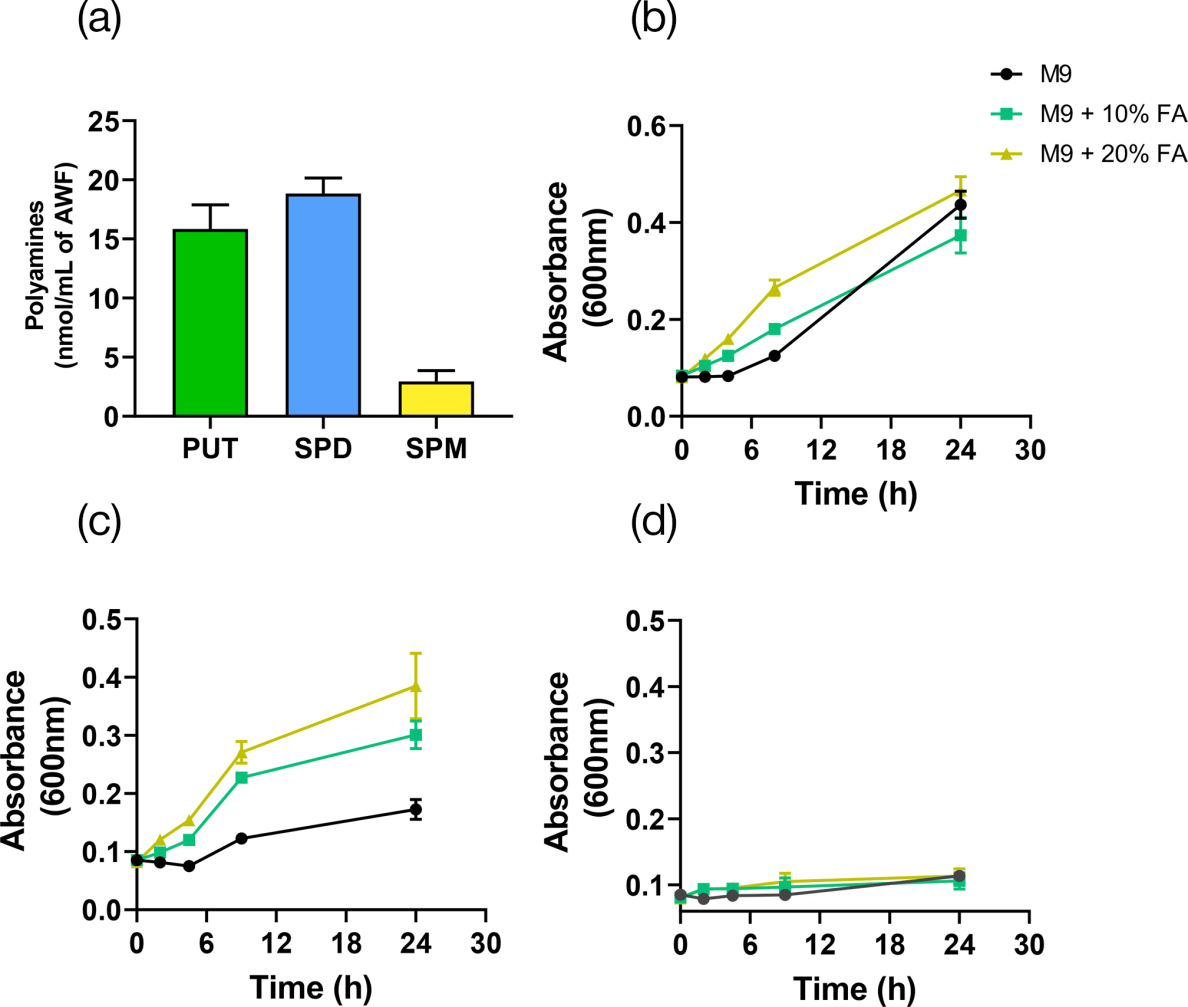
Effect of AWF supplementation on the growth of bacterial strains. (**a**) Quantification of polyamine contents in AWF extracted from *A. thaliana* leaves according to the methodology described in Methods. The growth of (b) *wt*, (c) *∆speE* and (d) *∆speA/speC* strains was evaluated in M9 medium supplemented with different concentrations of AWF. Growth was monitored at the indicated times by measuring the OD of the cultures at 600 nm. Bars designate sem. Each graph shows the result of one independent experiment. Each experiment was performed at least twice with similar results. PUT, putrescine; SPD, spermidine; SPM, spermine.

When examining these phenotypes, it is crucial to keep in mind that bacteria may be able to obtain and use plant-derived polyamines if their synthesis is prevented, as these are abundant in the apoplast (Fig. 7a). Therefore, we looked at bacterial growth in minimum medium M9 with and without the addition of AWF in order to explore the effect of plant nutrients. Results shown in Fig. 7(b) demonstrate that adding 10% or 20% AWF accelerated the exponential growth phase of the *wt* strain, even though the cell density at the stationary phase remained unchanged. This suggests that external metabolites, including polyamines, may enhance cell multiplication, but that polyamine synthesis provides sufficient levels of these compounds to achieve maximal cell density in minimal media. The growth of *ΔspeE* was stimulated in AWF-amended media in a dose-dependent manner and reached the growth levels shown by the *wt* strain with the addition of 20% AWF (Fig. 7c). In turn, the severe effects of *speA* and *speC* deletion in the *ΔspeA/speC* strain were not reversed by AWF, indicating the inability of this strain to incorporate polyamines from the external space (Fig. 7d). These findings imply that the acquisition of these molecules in the plant environment may serve as a partial compensatory solution to the spermidine deficit in the *ΔspeE* strain, contributing to the ability of this strain to colonize plant tissues. However, putrescine synthesis perturbation in *ΔspeA/speC* cannot be remedied by plant polyamines because of either ineffective uptake or incorporation or because their amounts are insufficient to support bacterial development.

## Discussion

Putrescine and spermidine have emerged as crucial regulators of bacterial physiological processes, significantly influencing pathogenesis and survival strategies. The production of polyamines by the host poses a major challenge in fully understanding their functions in pathogenic bacteria, as host-derived polyamines can obscure the true impact of bacterial gene deletions on the cytoplasmic concentrations of these compounds, potentially masking mutant phenotypes [38]. In order to address this and clarify the roles of polyamines in *Pto*, we evaluated *in vitro* multiple virulence-associated traits using mutants defective in polyamine biosynthesis and then studied their ability to invade plant tissues through phenotypic assessments in plants.

Initially, the capacity to control motility and the development of biofilms by polyamines was evaluated. As was previously indicated, switching between these states is an essential adaptive strategy for plant surface survival. Thus, while mobility aids in the quest for more suitable places and facilitates bacterial translocation towards the plant inner tissues, cell aggregation in the form of biofilms at the plant surface assists the bacteria to survive desiccation and other detrimental conditions [39,40]. These abilities are also very important for bacterial pathogens when growing endophytically, particularly for xylem-colonizing pathogens that actively move towards this space and occlude it with the formation of biofilms [41,42]. Recent data, however, indicate that the expression of flagellar genes in apoplastic pathogens like *Pto* is inhibited during host colonization [43]. This appears to be taking place in order to reduce bacterial detection by the plant defence apparatus and prioritize other advantageous virulence functions.

Remarkably, our earlier research revealed a negative association between the expression of important genes of the polyamine synthesis pathway and those involved in *P. syringae* flagellum development [16], suggesting that polyamines may be involved in controlling the transition of this pathogen between the motile and sessile stages. In agreement with this, we discovered in the present work that spermidine and putrescine influence bacterial motility. Disruption of spermidine synthesis promotes biofilm formation in M9, which appears to be linked to its effects on cell motility. Indeed, this mutation reduces swimming speed and the overall ability of the bacteria to swim, suggesting that reduced motility may favour the transition to a sessile, biofilm-forming state. In turn, motility and biofilm development diminish when there is no active putrescine synthesis pathway, which may be a consequence of reduced growth rate. Interestingly, we observed an increase in bacterial cell speed of the *ΔspeE* mutant when growing in the polyamine-rich medium LB. This suggests that polyamine synthesis is not necessary for movement in a high-nutrient context where the ability to incorporate environmental polyamines may render its *de novo* synthesis unnecessary.

These results are consistent with previous reports on the regulatory roles played by polyamines in motility and biofilm formation in other bacterial species. For instance, deletion of polyamine synthesis genes causes loss of swimming and biofilm formation in *D. zeae* [12]. Polyamine depletion also affects biofilm development in *Yersinia pestis* and *P. aeruginosa*, which is reversed by the addition of exogenous putrescine [31,44].

In turn, the increase in biofilm formation following disruption of spermidine synthesis was also demonstrated in *P. aeruginosa* [44]. In this case, a mutation in the *speD* gene (responsible for the production of the aminopropyl moieties required for the transformation of putrescine into spermidine) contributed to a sessile state. The question when analysing spermidine-deficient strains is whether their phenotypes are due to a reduction in spermidine levels or rather to the accumulation of putrescine. This answer remains still elusive, as neither *ΔspeD P. aeruginosa* nor our *ΔspeE Pto* strains displayed an increase in intracellular putrescine [14,44], which probably would be explained by a regulatory mechanism that adjusts the amount of this compound when its transformation into spermidine is occluded, either through regulation of biosynthetic enzymes, an increment in its export or its conversion into the non-protein amino acid gamma-aminobutyric acid. *Pto* is known to be able to assimilate putrescine as a nitrogen source. Therefore, more work should be conducted to demonstrate whether spermidine acts as a negative regulator of bacterial biofilm development or whether the acquisition of the sessile state is due to a positive role played by putrescine. In support of the latter, biofilm formation is promoted by the addition of putrescine to the culture medium of a *P. aeruginosa* mutant strain unable to catabolize this polyamine [44].

As in many other hemibiotrophic bacteria, *Pto* enters the plant tissue mainly via stomata [45]. Through the synthesis and secretion of the phytotoxin coronatine, this species has evolved the ability to prevent stomatal closure, a common response evoked in plants following pathogen recognition [33]. Our investigations demonstrated that the ability to inhibit stomatal closure in *A. thaliana* plants by the *ΔspeE* and *ΔspeA/speC* strains was significantly altered compared to the *wt* strain and comparable to the coronatine-deficient strain *corS*. Although it remains to be investigated whether these mutations lead to a deficiency in coronatine synthesis, it has been recently reported that polyamines can directly inhibit stomatal closure [46], providing an alternative explanation for the observed phenotype. This, however, seems to contradict our previous work showing that putrescine by itself does not affect stomatal conductance [15]. Therefore, it is worthwhile to investigate whether the ability to promote stomatal reopening of these strains is dependent on their capacity to secrete polyamines or the existence of a regulatory mechanism exerted by these compounds on the production of coronatine.

The production of siderophores capable of chelating iron such as pyoverdine constitutes another strategy that contributes to bacterial virulence, as its reincorporation ensures an iron supply [47]. Interestingly, the impairment of putrescine and spermidine synthesis in *ΔspeE* and *ΔspeA/speC* mutants led to diminished pyoverdine generation. Spermidine supplementation restored the ability of *ΔspeE* to synthesize pyoverdine, whereas putrescine did not increase the production of this compound in *ΔspeA/speC*, suggesting that spermidine is essential for siderophore synthesis. Barrientos-Moreno *et al.* [22] demonstrated that the production of the siderophore pyoverdine in *P. putida* is affected when arginine synthesis is interrupted, which can be overcome with the supplementation of the medium with spermidine.

Given that ornithine and arginine serve as precursors for pyoverdine synthesis in many pseudomonads, it would be logical to speculate a correlation between polyamines and the production of this siderophore [48]. However, *Pto* lacks PvdA, the ornithine hydroxylase responsible for the formation of *N*5-hydroxyornithine, and its pyoverdine is devoid of both arginine and ornithine-derived compounds [49]; consequently, the mechanism by which polyamine biosynthesis affects pyoverdine production in this strain remains unclear. This may pertain to the role of polyamines in regulating transcription and translation as well as enzyme activities [50].

It has been demonstrated that polyamines function as intracellular signalling molecules modulating the expression of the T3SS in different bacterial species [12,51, 52]. In agreement with this, our results demonstrated that mutations in *ΔspeE* and *ΔspeA/speC* contributed to a reduced expression of the T3SS-associated effector HopA (*ΔspeA/speC*) and a diminished induction of the HR in *N. benthamiana*. These results underscore the importance of polyamines in orchestrating bacterial virulence strategies, including effector secretion and host manipulation.

Taking all the aforementioned findings, it might be inferred that loss of putrescine and/or spermidine synthesis would compromise pathogenicity in *Pto*. In order to test this, we then assessed the ability of *ΔspeE* and *ΔspeA/speC* to cause disease in *A. thaliana*. We decided to evaluate both the flooding and the infiltration method for inoculation to weigh the importance of the different infection routes. The double mutant strain *ΔspeA/speC* exhibited a hypovirulent phenotype regardless of the inoculation method used, consistent with its reduced ability to secrete pyoverdine, form biofilms, swim and modulate stomatal closure. On the other hand, the *ΔspeE* mutant showed a reduced ability to multiply in plant tissues when flooding was used as the inoculation method but demonstrated similar virulence to the *wt* strain with the infiltration method. This could be explained by its reduced mobility and ability to inhibit stomatal closure, two factors that do not significantly impact disease development once the bacteria are inside plant tissues where host-derived spermidine is accessible. Indeed, this mutant showed a phenotype with increased biofilm production and tolerance to oxidative stress (which we previously reported at Solmi *et al.* [14]), two mechanisms that could provide it with better fitness compared to the *wt* strain.

**Fig. 8.**
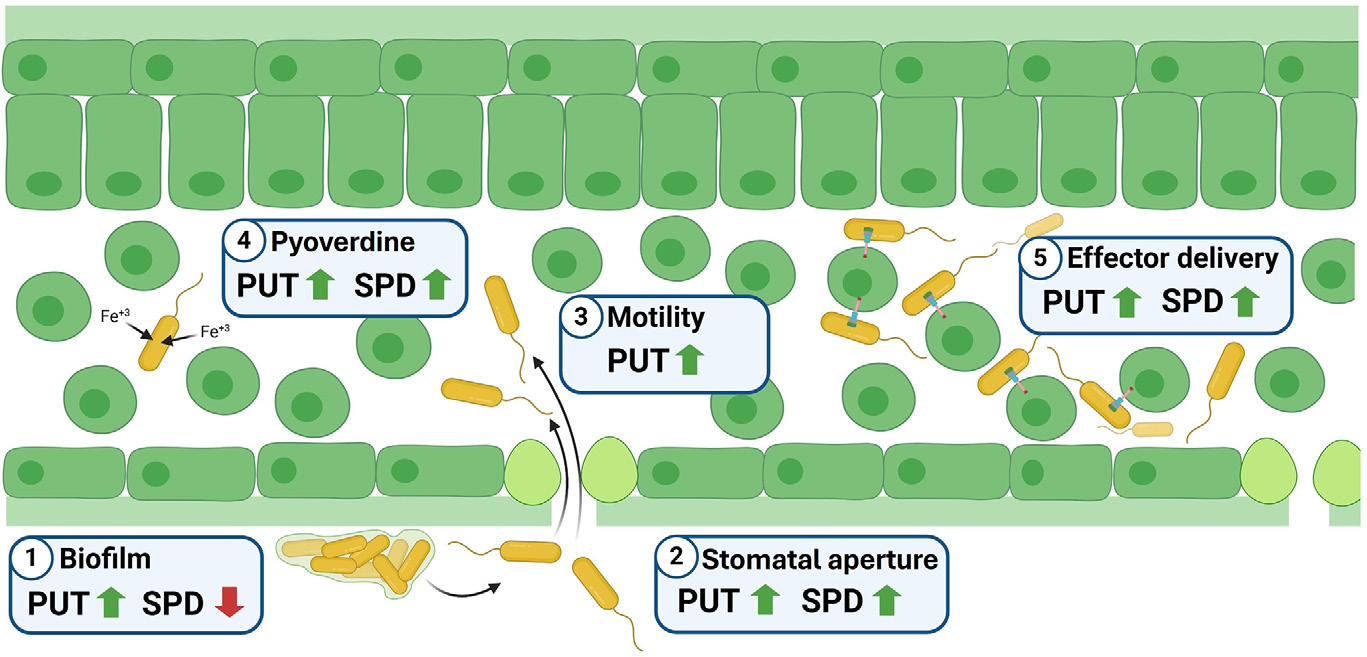
Scheme showing the potential influence of putrescine and spermidine biosynthesis on pathogenesis-related bacterial traits. (1) Biofilm production, important to resist harsh conditions found at the plant surface, seems to rely on an increase in putrescine production, whereas spermidine synthesis exerts a negative effect. However, both polyamines appear to have a significant positive influence on the capability of *Pto* to control (2) stomata aperture, (4) the production of pyoverdine and, therefore, the acquisition of iron and (5) the translocation of effectors into the host cell. (3) However, swimming motility seems to depend primarily on putrescine synthesis. Even though these results agree with the idea that blocking polyamine synthesis may have a dramatic impact on disease development, our research indicates that this is only true for putrescine production, as cells perturbed in spermidine synthesis are still able to retain virulence, which may be explained on the basis of the incorporation of this polyamine from plant tissues. PUT, putrescine; SPD, spermidine.

In summary, our research has demonstrated that a deficiency in polyamine synthesis significantly impacts various pathogenesis-related characteristics in *Pto* (Fig. 8). Thus, we found that putrescine production is essential for all virulence traits evaluated, including motility, biofilm production, stomatal control, iron acquisition and effector delivery. Spermidine synthesis demonstrated comparable roles, although in this case it seems to have a detrimental impact on biofilm formation and had no impact on swimming. Importantly, while polyamine biosynthesis is crucial for these processes, it does not necessarily alter bacterial virulence in a polyamine-rich plant environment. Therefore, further investigation is needed to understand the contribution of host-derived polyamines to bacterial virulence and multiplication in planta. This will require the inclusion of mutant strains deficient in polyamine transporters.

## Supplementary material

10.1099/mic.0.001569 Uncited Supplementary Material 1.

## Abbreviations

AWF: apoplastic washing fluids
DAB: 3,3-diaminobenzidine
HIM: Hrp-inducing medium
HR: hypersensitive response
T3SS: type III secretion system
