# Supplementary material for: The influence of the polyamine synthesis pathways on Pseudomonas syringae virulence and plant interaction
Source: Microbiology (Reading). 2025 Jun 10;171(6):001569. doi: 10.1099/mic.0.001569 (PMC12152252; doi:10.1099/mic.0.001569)
Supplement: Uncited Supplementary Material 1. [file mic-171-01569-s001.pdf]

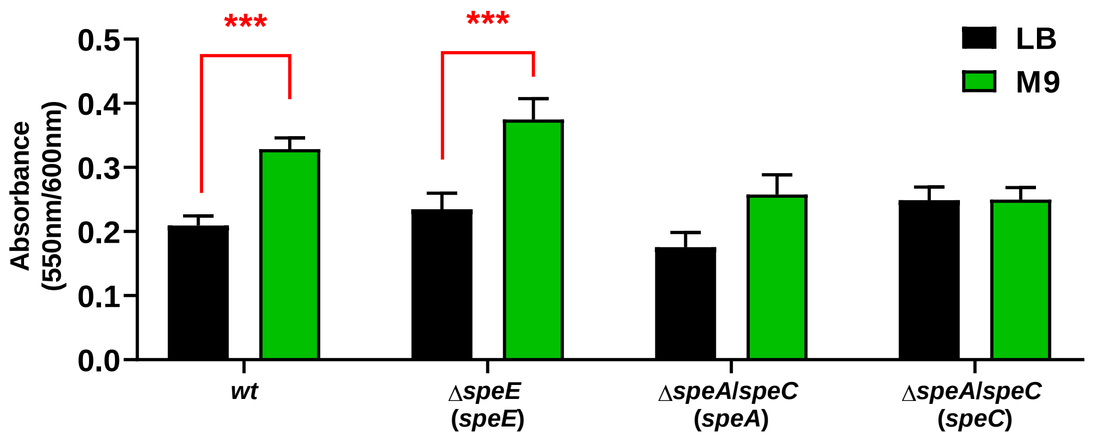


**Figure S1. The complementation of mutant strains restored biofilm formation to *wt* levels in polyamine synthesis mutants.** The formation of biofilms was quantified using the crystal violet staining method. Cells were grown statically in LB or M9 medium for 24 h at 22°C in multi-well plates. Subsequently, each well was washed, and staining performed as described in Materials and Methods. The absorbance at 550 nm was measured to estimate biofilm production, and values normalized to cell growth as determined by the absorbance at 600nm. Data were analyzed using ANOVA followed by Tukey's post hoc test for multiple comparisons. Significant differences between different growth media for the same strain are presented as ***$p < 0.001$. The assay was performed in triplicate with similar results.

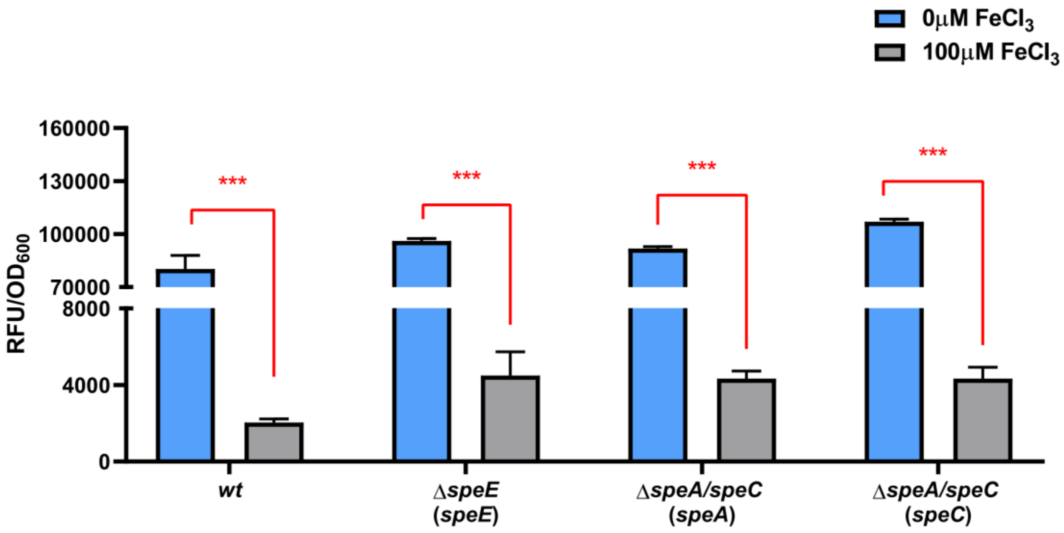


**Figure S2. The negative effects on pyoverdine production due to the lack of polyamine synthesis are reversed by complementation.** Cells were cultivated at 28°C for 16 h in M9 and M9 supplemented with 100 μM $FeCl_3$. The supernatants were collected by centrifugation and the Relative Fluorescence Units (excitation 398 nm/emission 455 nm) derived from pyoverdine were normalized to the estimated cell concentration as measured by $OD_{600}$.
